# Advances in Electroencephalography for Post-Traumatic Stress Disorder Identification: A Scoping Review

**DOI:** 10.1109/OJEMB.2025.3538498

**Published:** 2025-02-05

**Authors:** José A. Salazar-Castro, Diego H. Peluffo-Ordóñez, Diego M. López

**Affiliations:** Telematics Department and the Telematics Engineering Group (GIT)University of Cauca 28002 Cauca 190003 Colombia; College of ComputingMohammed VI Polytechnic University 479571 Ben Guerir 43150 Morocco; SDAS Research Group Ben Guerir 43150 Morocco; Faculty of EngineeringCorporación Universitaria Autónoma de Nariño 347449 Pasto 520001 Colombia; Telematics Department and the Telematics Engineering Group (GIT)University of Cauca 28002 Cauca 190003 Colombia; Health Sciences FacultyUniversity of Cauca 28002 Cauca 190003 Colombia

**Keywords:** Brain electrical activity analysis, diagnosis and therapy, electroencephalography, post-traumatic stress disorder, machine learning

## Abstract

*Background:* Post-traumatic stress disorder (PTSD) is a psychophysiological condition caused by traumatic experiences. Its diagnosis typically relies on subjective tools like clinical interviews and self-reports. *Objectives:* This scoping review analyzes computational methods using EEG signal processing for PTSD diagnosis, differentiation, and therapy. It provides a comprehensive overview of the entire EEG analysis pipeline, from acquisition to statistical and machine learning techniques for PTSD diagnosis. *Methods:* Using the PRISMA-ScR protocol, studies published between 2013 and 2024 were reviewed from databases including Scopus, Web of Science, and PubMed. A total of 73 studies were analyzed: 52 on diagnosis, 8 on differentiation, and 15 on therapy. *Results:* EEG Bands and Event-Related Potentials (ERP) were the dominant techniques. The Alpha band demonstrated strong performance in diagnosis and therapy. LPP ERP was most effective for diagnosis, and P300 for differentiation. Supervised SVM models achieved the highest accuracy in diagnosis (ACC = 0.997), differentiation (ACC = 0.841), and psychotherapy (ACC = 0.78). Random Forest multimodal models integrating EEG with other modalities (e.g., ECG, GSR, Speech) achieved ACC = 0.993. Unsupervised approach is employed to cluster patients to identify PTSD subtypes or to differentiate PTSD from other mental disorders. Veterans and combatants were the primary study population, and only three studies reported open datasets. *Conclusions:* EEG-based methods hold promise as objective tools for PTSD diagnosis and therapy. The review identified limitations in the use of ERP, sleep characterization and full-band EEG. Broader datasets representing diverse populations are essential to mitigate bias and facilitate robust inter-model comparisons. Future research should focus on deep learning, adaptive signal decomposition, and multimodal approaches.

## I. Introduction

Post-TRAUMATIC stress disorder (PTSD) significantly impacts quality of life. Direct or indirect exposure to traumatic events (e.g., war, violence, and sexual abuse) is a key factor in the development of stress-related disorders, with PTSD being the most prevalent [1], [2]. While up to 90% of the population may experience trauma, only 20–30% develop PTSD [3]. The World Health Organization (WHO) reports that 6% of individuals experience PTSD at some point in their lives [4]. Despite its prevalence, PTSD remains underdiagnosed [5].

PTSD, initially classified as a raw stress response in DSM-1 and recognized as a posttraumatic stress disorder in DSM-3 (1980), faces diagnostic challenges due to evolving criteria and definitions [3], [6], [7]. Comorbidities like major depressive disorder (MDD) and traumatic brain injury (TBI) further complicate identification and treatment [3], [8].

The DSM-5 and ICD-11 highlight PTSD symptoms such as intrusion, avoidance, and cognitive and mood alterations, with DSM-5 also including hyperarousal [9]. Notably, DSM-5 defines PTSD as a single category with subtypes, while ICD-11 separates it into PTSD and Complex PTSD (CPTSD) [10], [11]. DSM is recognized for a broader diagnostic scope, whereas ICD is valued for clinical utility.

### A. Rationale

PTSD diagnosis predominantly relies on subjective assessments through clinical interviews and questionnaires, such as the Clinician-Administered PTSD Scale (CAPS), Primary Care PTSD Screen (PC-PTSD), and the Posttraumatic Stress Disorder Checklist (PCL). This process is subjective and influenced by the clinician's expertise [12], [13]. Identifying reliable diagnostic biomarkers is essential to improve accuracy, objectivity, and treatment efficacy [14]. Biomarker research has focused on neuroimaging techniques like functional magnetic resonance imaging (fMRI), magnetoencephalography (MEG), and electroencephalography (EEG) [15], [16].

EEG stands out for its non-invasive nature, lower cost, portability, and high temporal resolution [17]. Common EEG-based biomarker methods include full-band EEG analysis, frequency band analysis (e.g., alpha (α), beta (β), gamma (γ), theta (θ), delta (δ), and sigma (σ) rhythm), event-related potentials (ERP), and sleep stage analysis (REM and non-REM). Despite advancements, challenges remain in understanding and enhancing current EEG analysis, characterization, and classification techniques [18], [19].

Previous reviews on EEG and PTSD biomarkers [16], [20], [21], [22], [23], [24], [25], [26], [27] have limitations, excluding diverse biomarkers and mental health application contexts such as diagnosis, differentiation, and therapy. Additionally, they lack comprehensive evaluations of machine learning approaches—including supervised, unsupervised, and multimodal methods—limiting comparisons and applications.

### B. Objective

This scoping review aims to analyze EEG computational methods for PTSD diagnosis, differentiation, and therapy. It provides a comprehensive overview of the methods applied across the entire EEG analysis pipeline, from data acquisition and preprocessing to statistical and machine learning techniques and explores potential future directions for improving PTSD management.

## II. Methods

The review follows the PRISMA-ScR checklist [28], with key reporting items detailed in this section. A full description of the items is provided in the Supplementary Materials.

### A. Eligibility Criteria

This review included studies describing EEG-based solutions for PTSD in human patients. Eligible studies analyzed EEG signals for diagnosis (vs. healthy controls), differentiation (vs. other disorders), or treatment response. Articles in English published from January 1, 2013, to June 30, 2024, were considered. Participants had to have experienced trauma and been diagnosed or identified as probable PTSD cases using validated clinical tools. Excluded were studies involving other neuroimaging techniques, animals, or participants under 7 years old. Experimental studies and case reports were included, while reviews, meta-analyses, and theoretical studies meeting inclusion criteria were classified as secondary and used to contextualize results.

### B. Information Sources and Search

Scopus, Web of Science (WoS), and PubMed databases were queried using the search string: ("Post-Traumatic Stress Disorder" OR "Post Traumatic Stress Disorder" OR "Posttraumatic Stress Disorder" OR "PTSD") AND ("EEG" OR Electroencephalogra*) AND ("Computer-Assisted Diagnosis" OR "Computer Assisted Diagnosis" OR diagnos* OR identif* OR "differentiation" OR "machine learning" OR "automatic classification"). The strategy was adapted to each database using MeSH terms, with the publication period limited to January 1, 2013–June 30, 2024, and searches focused on titles, abstracts, and/or keywords.

### C. Data Charting Process

A data extraction template was used to gather detailed information from eligible sources, available in the supplementary materials. The template items included 1) title, 2) author(s), 3) year of publication, 4) abstract analysis, 5) type of study, 6) mental health application context, 7) type of brain activity analysis (EEG bands, ERP, sleep characterization or full-band EEG), 8) specific EEG bands or ERP (if applicable), 9) study population, 10) comparison group, 11) type of EEG model/technique analysis and 12) evaluation metrics.

## III. Results

**Fig. 1.**
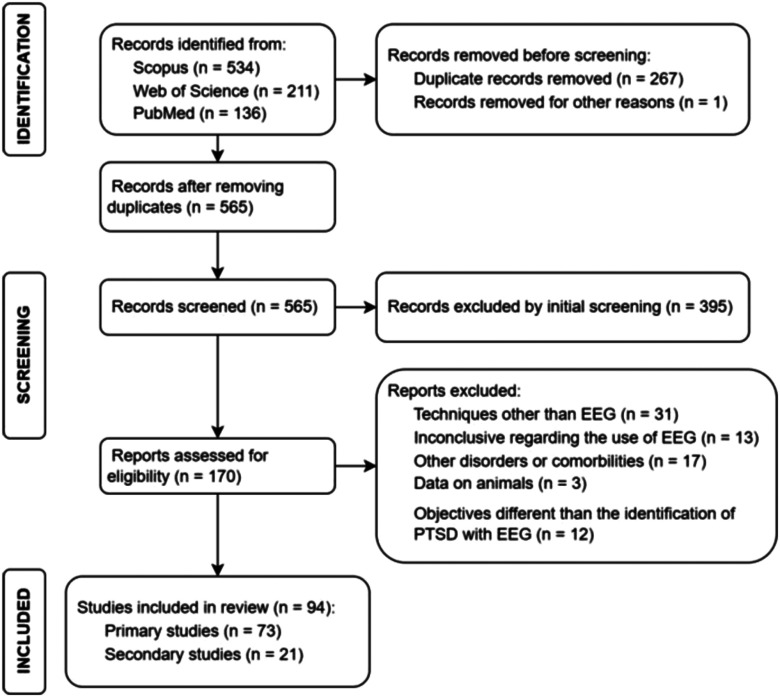
Flowchart illustrating the selection process from 795 studies to the 86 studies to be reviewed.

A total of 73 documents were considered eligible for this scoping review and the relevant data extracted from each source is provided in the supplementary materials section. Fig. 1 presents a flowchart illustrating the study selection process.

First, a bibliometric analysis was performed to identify the main topics and research trends. Subsequently, according to the PRISMA-ScR guidelines, primary studies were analyzed in detail, considering the topics identified in the previous data charting process.

### A. Bibliometric Analysis

The bibliometric analysis was supported by the R Bibliometrix package and reported in the supplementary material. This analysis considered the trends in the number of studies published per year, the most relevant authors in the field, the categorization of topics by relevance and development, a co-occurrence analysis and a systematic map that summarizes the literature found.

**Fig. 2.**
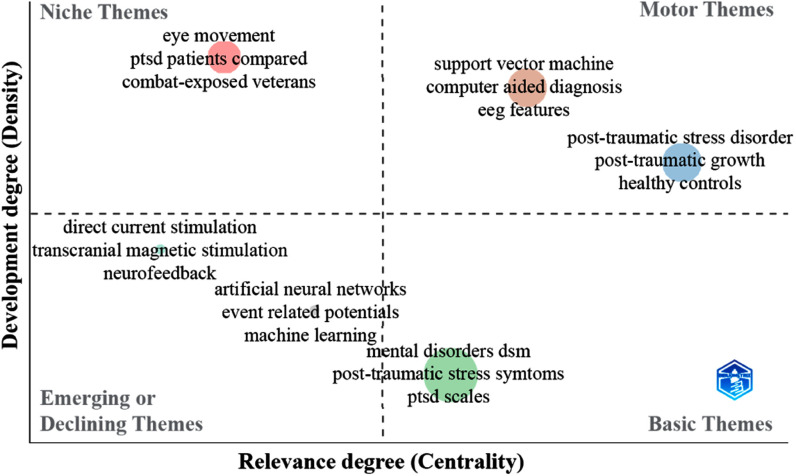
Categorization of the main themes found in the documents through a thematic distribution map considering the degree of development and relevance.

Fig. 2 presents a thematic distribution map, categorizing research topics based on their relevance and progression. The basic themes quadrant provides a foundation for understanding fundamental research areas, such as PTSD symptoms, scales, and other DSM-based disorders. Niche themes, like eye movement sleep characterization, delve into more specialized and emerging research areas. The motor themes reveal robust research areas, such as the use of SVM for identifying PTSD and other disorders. This prompts us to consider the study of comorbidities in a PTSD differentiation scenario, such as major depressive disorder (MDD). Emerging themes focus on the mental health context of treatments, indicating a growing interest in the use of EEG for identifying PTSD using machine learning models and artificial neural networks, or evaluating the response to treatments like (direct-current stimulation) DCS, transcranial magnetic stimulation (TMS) or neurofeedback.

**Fig. 3.**
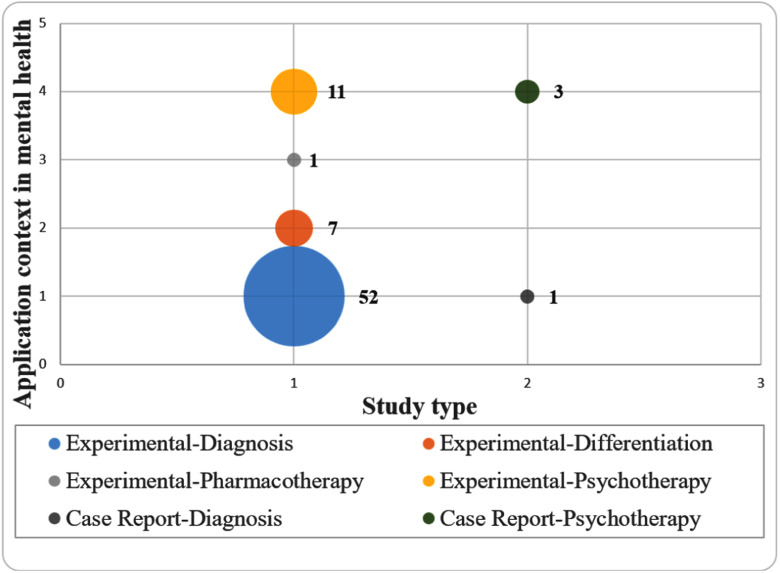
Systematic mapping considering the type of research (horizontal axis: 1**-**Experimental and 2**-**Case Report) and the context (vertical axis: 1**-**Diagnosis, 2**-**Differentiation, 3**-**Pharmacotherapy, and 4**-**Psychotherapy).

Fig. 3 shows a systematic map representing the areas that have been most studied (biggest circles) establishing relationships between the type of study and the mental health context. Overall, most of the primary studies are experimental studies (n = 69, 94.52%) and the remaining are case reports (n = 4). These experimental studies are distributed in four categories describing the application context in mental health: 73.24% are in the category PTSD diagnosis (n = 52), 7.69% of the studies are in the category of differentiation from other disorders (n = 7), 15.49% are in the category of identification of PTSD in psychotherapy (n = 11), and only 1 study is in the category of pharmacotherapy. Since [30] and [63], two of the 73 studies reported results in both diagnosis and differentiation, they were included in both applications. Thus, the total number of papers on the map is 75.

### B. Scoping Review Synthesis

We used the template described in Section II-C as a data chart to synthesize the main findings. We classified the findings into four topics: 1). Type of EEG-based Brain Electrical Activity Analysis, 2). Study population and comparison groups, 3). Available data sets, and 4). Type of EEG analysis model/technique.

#### 1) Type of EEG-Based Brain Electrical Activity Analysis

**Fig. 4.**
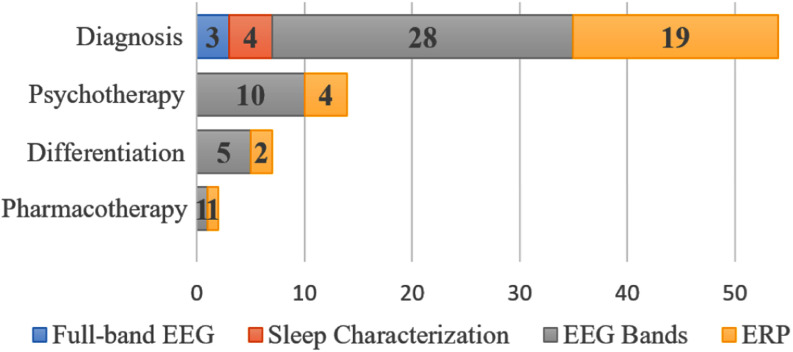
Type of EEG-based brain electrical activity used in the studies considering the four application contexts in mental health.

We classified the brain electrical activity analysis (BEAA) techniques into four categories: full-band EEG, sleep characterization, EEG bands, and ERP. This analysis was conducted for the four application contexts in mental health: diagnosis, differentiation, psychotherapy, and pharmacotherapy. Fig. 4 shows the number of BEAA techniques in each one of the four contexts.

In general, all BEAA techniques are used in all four application contexts in mental health. As can be seen from the sum of the values, in the same way that a study can address different application contexts, a study can also use more than one BEAA technique and would be scored for each one.

All four BEAA approaches have been used in PSTD diagnosis, but EEG bands and ERP techniques are the more frequent ones, accounting for 51,85% (n = 28) and 33,3% (n = 18) studies, respectively. In differentiation and therapy, only ERP and EEG bands have been implemented, being EEG bands the most frequent one.

The analysis was complemented by characterizing each one of the four types of BEAA techniques. Table I defines each technique and summarizes the findings and the more relevant studies. The supplementary material presents a more detailed analysis of each one of the techniques.

**TABLE I:** Overview of Different Types of Brain Electrical Activity Analysis Used for PTSD

**Brain Electrical Activity Analysis**	**Description**	**Findings**
EEG Bands (See S-Table V)	Whole EEG is decomposed into different frequency bands (Alpha, Beta, Gamma, Delta, Theta, and Sigma) or specific frequency ranges. These bands are characterized, allowing identification between patients with and without PTSD by statistical evaluation of differences between features or through ML models.	The most used bands for the identification of PTSD are Alpha (n = 38), Beta (n = 36) and Theta (n = 34), with Sigma (n = 5) and the full-band EEG (n = 2) being the least explored. Standard filters, fixed segments or CWT are used for decomposition. No studies were found that involve more novel techniques such as multivariable adaptive signal decomposition. Studies indicate that Alpha band provides better results in diagnosis and psychotherapy, and Beta in differentiation. Gamma is the only band explored in all four contexts. ML models showed that all bands are informative, the discriminating factors are given by the features extracted from the bands.
ERP (See S-Table VI)	ERP are specific brain responses triggered by a stimulus and are measured as variation in the electrical activity of the brain at a time following the stimulus. The ERP features analyzed are the variations that represent abnormal patterns between patients with and without PTSD.	In diagnosis, a wide variety of ERP have been used, with LPP being the most frequently utilized and yielding the best results for identifying PTSD. P3 and VPP are also showing promising outcomes. ERP such as EPN, N100, N200, and NSW were studied only once and did not demonstrate significant changes in diagnostic applications. In differentiation, the P300 ERP has shown the most effective results. In psychotherapy, P3 is the most studied ERP and, along with FRN, and LPP. In the only pharmacotherapy study, ASS, MMN and P50 were used and yielded good results. However, in differentiation and psychotherapy, no study has explored more than one ERP simultaneously, which limits the ability to compare and determine which ERP provides the best results.
Sleep Characterization (See S-Tables III and IV)	It focuses on identifying different states (awake or asleep) between patients with and without PTSD, and sleep stages among NREM or REM patients. Characteristics related to amplitude, frequency, or duration of sleep spindles (rapid oscillations characteristic of NREM) show variations among participants. The number of studies in this BEAA are limited.	The key features calculated for REM and NREM states are sleep latency, sleep efficiency, stage 1 NREM percentage, stage 2 NREM percentage, SWS percentage, REM percentage and REM latency. Only one study concluded that features related to the number of awakenings and the percentage of wakefulness facilitate the identification of PTSD when analyzing the awake state. They also determined to characterize the NREM states N1 and N2, and these were the ones that presented the best results considering the N3 state. Two studies consider sleep spindles, FS and SS. The features extracted from these oscillations are amplitude, duration, oscillatory frequency, density, PLV and MPD. One of them mentions that the frequency features of both spindles are more differential in better identifying PTSD patients. The second study mentions that MPD performs better than PLV in SS. The latter features did not present important differences in FS. Only one study out of four obtained better results when characterizing REM. It is evident that the results depend on the features extracted for comparison.
Full-band EEG (See S-Table II)	It involves the whole EEG signal, preprocessed with filters and without artifacts, but without decomposing. The analysis is performed by individual channels or by the connectivity between ROIs. Extracted features, e.g., Hurst exponent for each channel, or features at source or sensor level, show different values ​​between patients with and without PTSD. ML models are also used.	Due to the non-stationary nature of EEG signals, all studies segmented the signal with different time window sizes, but no standardized size was reported. Only one study compared windows ranging from 5 to 60 s, concluding that segmentation is useful for data augmentation. However, overlapping can introduce redundancy and overfitting due to data correlation. No modern preprocessing or segmentation methods other than the standard ones are evident. To extract features from the whole EEG and compare values between patients, Hurst analysis (a long-range correlation measure in EEG) [32], or CWT to each segment to obtain time-frequency maps [33] or the use of CNN to extract features from the last layer of EEGNet (optimized for EEG data) [36] were demonstrated.

EEG: Electroencephalography; ERP: Event related potentials; REM: Rapid eye movement; NREM: No REM; LPP: Late Positive Potential; VPP: Vertex positive potential; EPN: Early posterior negativity; NSW: Negative slow wave; FRN: Feedback-related negativity; ASS: Auditory Stady State; MMN: Mismatch negativity; FS: Fast spindles; SS: Slow spindles; PLV: Phase-locking value; MPD: Mean phase difference; CWT: Continuous wavelet transform; CNN: Convolutional neural network; ROI: Region(s) of interest; ML: Machine learning.

#### 2) Study Population and Comparison Group

**Fig. 5.**
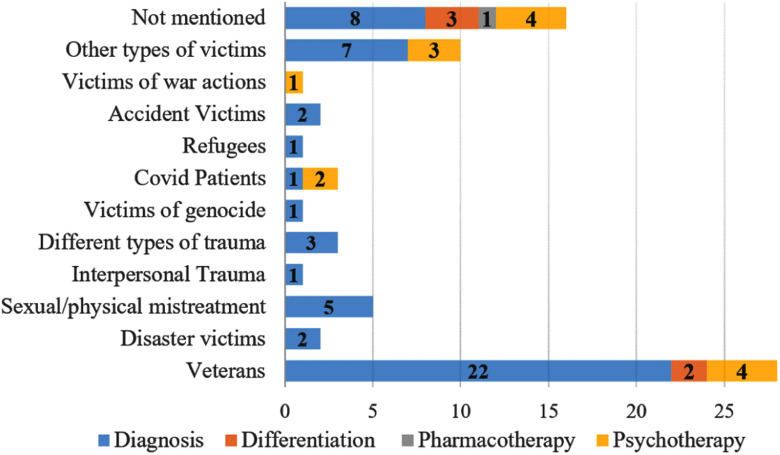
Studied population in the different analyzed documents according to the application context in mental health.

Analysis in Fig. 5 shows that 38.36% of the studies focused on veteran and combatant populations (n = 28). Diagnosis was the most frequent application context and included studies in various population groups. Psychotherapy was the second most common context. In the single pharmacotherapy study included, the type of patients involved was not reported.

For a more detailed analysis of the population presented in the different studies, we grouped them into a) studies including only patients with PTSD, b) studies including a control group of healthy patients, and c) studies that include patients with other disorders as a control group. For each group, the number of participants, the gender, age, distribution between the control group and the type of group was summarized in the S-Table VII, available in the supplementary material section.

#### 3) Available Datasets

Table II describes the available datasets. For each dataset we present the application context for which they were used, the year of publication, the number of EEG channels, the number of participants and the type of population (PTSD, other disorders (OD) or healthy controls (HC)). We also present the age and gender distribution of participants.

**TABLE II:** Summary of Open Datasets

**Study/Year**	**Application in Metal Health**	**Ch**	**Participants per Group**	**Characteristics of Participants**
[32] 2018	Diagnosis	31	6 PTSD/6 HC	Males 27 ± 5 years
[66] 2021	Differentiation	19	945 PTSD/OD/HC	Male and Female See S-Table VIII
[98] 2022	Psychotherapy	832	20 PTSD	Females 53.72 ± 6.1 years

Ch: Channels; PTSD: Post-traumatic stress disorder; HC: Healthy controls

Only three studies with open EEG data/signals were identified. In diagnosis application, the dataset included 6 PTSD and 6 healthy participants, not intended for machine learning applications. In differentiation, the dataset comprised EEG-derived features and demographic data (e.g., gender, age, education, IQ). Its relevance lies in the number of participants and the fact that they have a wide variety of disorders detected, including a group of healthy patients -which could indicate that it may be useful in ML models for PTSD diagnostic and differentiation. Finally, in psychotherapy, the dataset encompassing 32-channel EEG recordings from 20 PTSD patients pre- and post-treatment, with the responses to PTSD questioners such as PCL, PC-PTSD, Harvard Trauma Questionnaire (HTQ), before and after each neurofeedback session.

#### 4) Type of EEG Analysis Model/Technique Synthesis

**Fig. 6.**
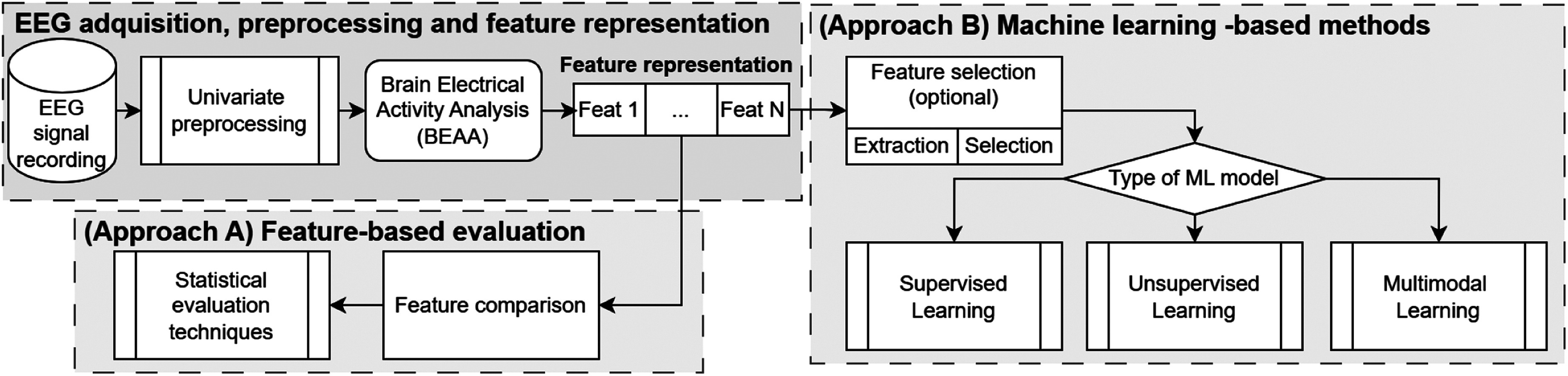
General data pipeline used in the included studies. The first steps include EEG acquisition, signal preprocessing and signal feature representation. Next, two approaches are used to identify PTSD: feature comparison with statistical evaluation techniques or machine learning methods.

Although the EEG recording, preprocessing, and analysis protocol depend on the type of BEAA used, we present an overview of the signal processing pipeline for identifying PTSD through EEG analysis based on the included studies, as illustrated in Fig. 6. The EEG acquisition phase adheres to the 10/20 system for electrode placement, with the number of electrodes determined by the specific study design. During pre-processing, univariate filters are applied to each channel to mitigate noise and confine the signal within a frequency range spanning from 0 Hz to 40–100 Hz. Segmentation is commonly employed, particularly in studies that analyze the full EEG spectrum or decompose signals into specific frequency bands. Pre-processing concludes with artifact removal, addressing interference from ocular, muscular, and cardiac activities [99].

Despite these advancements, most studies rely on standard techniques, such as univariate filters, wavelet transforms, or Fourier analysis, for signal conditioning, decomposition, and feature representation. However, the non-linear, non-stationary, and complex nature of EEG signals limits the efficacy of these methods, as they often exhibit suboptimal time-frequency localization. Adaptive univariate techniques, such as Empirical Mode Decomposition (EMD), Variational Mode Decomposition (VMD), and Iterative Filtering (IF) address this challenge [100]. While these methods enhance signal analysis, univariate processing risks the loss of mutual information across EEG channels [101]. Consequently, emerging approaches emphasize adaptive multivariate decomposition techniques, such as Multivariate Iterative Filtering (MIF), which address these limitations and preserve inter-channel relationships [102].

The final stage of PTSD identification diverges into two primary approaches: feature-based evaluation and machine learning (ML) models. Feature-based evaluation involves statistical analyses of extracted BEAA features to compare variations across patient groups, such as differences between control and PTSD groups. Conversely, ML approaches use supervised methods to classify individuals as either healthy or PTSD-diagnosed, or as responders versus non-responders to therapy. Unsupervised methods cluster PTSD subtypes, symptoms, or comorbid disorders. Multimodal approaches, yet underexplored in prior EEG-based PTSD reviews, combine diverse data modalities—including sketches, electrocardiography (ECG), head movements, galvanic skin response (GSR), and speech.

### C. Type of EEG Analysis Model/Technique

#### 1) Feature-Based Evaluation Studies

Common statistical techniques employed are hypothesis and statistical significance tests, used to determine if there is a significant difference between the groups or variables being compared. Results reported include hypothesis tests such as t-test, Wilcoxon rank-sum, Mann-Whitney U and Wilcoxon signed-rank. Furthermore, some studies consider the Z-score as a measure of deviation. Other techniques used are those focused on measuring and analyzing the variance between two or more groups (in the case of differentiation). Results include analysis of variance (ANOVA) or its variations such as repeated measures analysis of variance (RANOVA), analysis of covariance (ANCOVA) and multivariate analysis of variance (MANOVA). Also, other techniques measure the associations between two variables using Spearman or Pearson correlations. Finally, some studies use regression models such as linear, hierarchical, and mixed models. These models help to decide which characteristics best predict the presence of PTSD or symptomatic severity, allowing them to quantify the impact of each predictor.

#### 2) Unsupervised Machine Learning Studies

Unsupervised machine learning (USL) models are employed in three diagnosis and one differentiation studies, which are described in Table III. The USL K-means in diagnosis groups patients into clusters to identify PTSD subtypes or symptomatic groups. In differentiation, Agglomerative hierarchical clustering has been used to cluster participants with different disorders. The Calinski-Harabasz (C-H) index was used to assess how disperse the clusters and the data within clusters are. In addition, [19] and [65] considered the Gap metric to determine the optimal number of clusters but only [65] reports the results. [19] uses USL as a pre-training stage to improve the performance of SL classifiers by considering a separation of the PTSD group into subgroups according to subtypes.

**TABLE III:** Unsupervised Learning Approaches Used to Identify PTSD and Its Metrics

**Study/Year**	**Context**	N° of Channels and bands*	Participants**	**Model**	**Feature(s)**	**C-H**	**GAP**
[52] 2017	Diagnostic	32 **Alpha** band	55 PTSD females 14.07±1.13	K-means with 2 clusters	Frontal Asymmetry	40.17	-
[27] 2021	Diagnostic	26 Alpha, **Beta**, Gamma, and Theta bands	106 PTSD/95 HC 96M, 10F /85M, 10F 34.1±7.6/32.6±8.1	K-means with 2 clusters	Power envelope-based functional connectivity	50	-
[19] 2022	Diagnostic	22, 31 and 19 **Alpha**, Beta, Gamma, Theta and Delta bands	See Participants in Table III, study [19]	Sparse k-means with 2 clusters	Source functional connectivity	-	✓
[65] 2018	Differentiation	32 Alpha, **Beta**, Theta, and Delta bands	420, See S-Table VIII 164 M, 256 F 39.8 ±14.1	Agglomerative hierarchical clustering with 6 clusters	Power-based features	170	0.72

C-H: Calinski-Harabasz.

*The band that shows better results is highlighted in bold.

**It presents participants per group, followed by the distribution between males (M) and females (F), and the mean age and standard deviation.

#### 3) Supervised Machine Learning Studies

Supervised machine learning models (SL) are employed in PSDT diagnostic (n = 14), differentiation (n = 4) and psychotherapy (n = 2) contexts. No studies in pharmacotherapy were found. We compare the different SL studies in three tables describing the participants and their demographics, the type of BEAA, the ML model, the extracted features, and the best reported performance metrics.

Table IV describes SL approaches used in PTSD diagnosis. Support vector machine (SVM) is the most used method, considered in 10 of the 14 studies. Deep learning approaches are also evident in [36] and [59], and a transfer learning VGG16 model is deployed in [33]. Unlike other ML approaches, in EL a wide range of features are explored. Features analyzed are obtained from frequency and time domains, statistical measurements, and morphology of the signal. Methods with best accuracy (ACC) are SVM (0.997) and EEGNet (0.999). Table IV shows a comparison of other performance metrics and their average values. There is only one study involving ERP, with P300 [30]. [33] and [36] analyze the full-band EEG. The remaining consider the different EEG bands. No studies on sleep characterization were found. In [35] an interesting FgMDM classifier based on Riemannian geometry is presented with better results than other classifiers, including SVM.

**TABLE IV:** Supervised Learning Approaches Used to Identify PTSD in Diagnostic Contexts and Its Metrics

**Study/ Year**	N° of Channels and BEAA*	PTSD/HC Participants**	**Model(s)**	**Feature(s)**	**ACC**	**AUC**	**PPV**	**Sens**	**Spec**	**FScore**	**CV**
[30] 2019	64 **P300** ERP	28/39 14 M, 14F/18M, 21F 43.50±8.88/38.74±9.05	Linear SVM	Amplitude and latency in source and sensor levels	0.821	-	-	0.795	0.857	0.61	20 folds
[7] 2020	64 Alpha, Beta, Gamma, and **Theta** bands	106/95 96 M, 10 F/85 M, 10 F 34.1±7.6/32.6±8.1	Linear SVM	Power envelope connectivity in source	-	0.898	-	0.802	0.849	-	LOOCV
[29] 2020	64 Alpha, Beta, Gamma, Delta, **Theta,** and Specific bands	25/25 13 M, 12 F/9 M, 16 F 42.68±8.47/42.08±7.87	Linear SVM	Global/local network indices and nodal clustering coefficients	0.8	-	-	-	-	-	LOOCV
[35] 2020	62 **Alpha**, **Beta**, **Gamma**, **Delta**, **Theta,** and **Specific** bands	42/39 5 M, 37 F/8 M, 31 F 40.12±11.07/41.15±12.31	FgMDM	Spatial Covariance Matrices (SCMs), power and global/nodal indices	0.731	0.797	-	0.687	0.771	-	10 folds
			LDA		0.64	0.536	-	0.717	0.556	-	
			SVM radial		0.646	0.64	-	0.75	0.533	-	
			Random Forest		0.62	0.664	-	0.679	0.556	-	
[39] 2020	64 **Alpha**, Beta, **Gamma**, **Delta**, Theta, Sigma, and Specific bands	31/47 All males 31.3±4.7/32.8±6.2	Logistic Regression	Power Spectral Density (PSD) and synchrony in phase	-	0.9	0.31	**0.85**	0.67	-	6 folds
[57] 2021	64 **Beta**, Delta and, **Theta** bands	77/58 28 M, 49 F/30 M, 28 F 40.92±11.93/39.98±11.63	Linear SVM	Sensor/source network indices and Phase Locking Value (PLV)	0.704	0.85	-	0.727	0.672	-	LOOCV
[19] 2022	22, 31 and 19 **Alpha**, **Beta**, Gamma, Delta, and **Theta** bands	26/26 (22 channels) 35.42±8.24	Linear SVM	Spectral, long-range temporal correlations, k-means microstates and source functional connectivity features	0.629	0.68	-	0.646	0.612	-	10 folds
		44/33 (31 channels) 39.83±9.53	LR with sequential forward selection		0.66	0.92	-	0.66	**0.928**	-	
		61/30 (19 channels) 35.11±8.59	Random Forest		0.631	0.66	-	0.648	0.613	-	
[33] 2022	16 **Full-band EEG**	15/30 All males 52.82±4.283/51.90±2.95	VGG16 (Pre-trained CNN)	Frequency-time maps	-	0.789	-	-	-	-	15 folds
[46] 2022	19 **Alpha**, **Beta**, **Delta**, **Theta,** and **Specific** bands	61/61 25 M, 36 F/14 M, 47 F 41.1±13.1/40.9±9.7	Linear SVM	Spatiotemporal features	0.76	0.75	-	0.79	0.74	-	10 folds
[58] 2022	64 Alpha, Beta, Gamma, **Delta**, and **Theta** bands	Same that [57]	Linear SVM	PSD in source	0.866	**0.93**	-	-	-	-	10 folds
			Shallow ConvNet		0.731	-	-	-	-	-	
			EEGNet		0.761	-	-	-	-	-	
			13-Layer CNN		0.77	-	-	-	-	-	
[36] 2023	64 **Full-band EEG**	Same that [57]	CNN-13 segment windows of 5, 10, 15 and 20 seconds	Extracted with CNN	0.742 0.815 0.796 0.856	- - - -	- - - -	- - - -	- - - -	- - - -	10 folds
			EEGNet segment windows of 5, 10, 15 and 20 seconds		0.709 0.801 0.981 **0.999**	- - - -	- - - -	- - - -	- - - -	- - - -	10 folds
[59] 2023	8 or 32 **Alpha** and **Beta** bands	9 PTSD All females Not mentioned	Linear SVM	Statistics, frequency and morphology features	**0.997**	-	**0.99**	-	-	**0.99**	6 folds
			ANN		0.978	**-**	**-**	**-**	**-**	**-**	
[60] 2023	64 **Alpha**, Beta, **Gamma**, Delta, and Theta bands	30 HPTS /30 LPTS Not mentioned	Linear SVM	PSD	0.925	-	-	-	-	-	5 folds
[62] 2023	19 **Alpha**, Beta, Gamma, **Delta**, and Theta bands	5/5 Not mentioned	Histogram GB Light GB Machine	Spectrogram-based features	0.757 0.756	0.70.7	- -	- -	- -	0.83 0.73	-
				**Average**	**0.782**	**0.761**	**0.65**	**0.729**	**0.696**	**0.79**	**-**

BEAA: Brain electrical activity analysis; HC: Healthy control; HPTS: High PTSD symptoms; LPTS: Low PTSD symptoms; FgMDM: Fisher geodesic minimum distance to the mean; LDA: Linear Discriminant Analysis; LR: Logistic Regression; CNN: Convolutional Neural Network; GB: Gradient Boosting; ACC: accuracy; AUC: area under the curve; PPV: positive predictive value; Sens: sensibility; Spec: specificity; CV: cross validation; LOOVC: Leave one out cross validation.

*The band(s) or ERP that show(s) better results are highlighted in bold.

**It presents participants per group, followed by the distribution between males (M) and females (F), and the mean age and standard deviation.

Table V describes four SL studies in PTSD differentiation. The most studied classifier has been SVM. In terms of accuracy, SVM and C5.0 present the best results with 0.841 in both models. Features such as amplitude and power are the ones that give the best results, specifically considering the analysis with EEG bands. In [30] and [93], the ERP P300 is analyzed in differentiation between PSTD and MDD patients. The remaining two studies analyze the EEG bands and consider disorders such as bipolar disorder (BD), attention deficit hyperactivity disorder (ADHD), MDD, obsessive-compulsive disorder (OCD), opioid disorder, schizophrenia, mood disorders, acute stress disorder and adjustment disorder.

**TABLE V:** Supervised Learning Approaches Used to Identify PTSD in Differentiation Contexts and Its Metrics

**Study/ Year**	**N° of Channels and bands/ERP**	Participants**	**Model(s)**	**Feature(s)**	**ACC**	**AUC**		**PPV**		**Sens**		**Spec**		**FScore**		**CV**
[30] 2019	64 **P300** ERP	28 PTSD/67 MDD 14 M, 14 F/24 M, 43 F 43.50±8.88/42.09±9.83	Linear SVM	Amplitude and latency in source and sensor levels	0.71	-		-		0.806		0.5		-		LOOCV
[66] 2021	19 Alpha, **Beta**, Gamma, Theta, and Delta bands	52 PTSD/798 in nine specific diagnoses/95 HC See S-Table VIII	Linear SVM	PSD and Functional Connectivity	-		0.86		-		-		-		-	10 folds
			RF		-		0.87		-		-		-		-	
			LR (with EN penalty)		-		**0.877**		-		-		-		-	
[93] 2022	64 **P300** ERP	30 PTSD/30 MDD 15 M, 15 F/9 M, 21 F 42.77±11.16/44.60±10.08	Linear SVM	Average amplitudes	0.733	-		-		0.9		0.567		-		LOOCV
[67] 2023	19 **Alpha**, **Beta**, **Theta** and **Delta** bands	146 PTSD/404 in six specific diagnoses/84 HC See S-Table VIII	C5.0	Absolute power	**0.841**		-		0.757		**0.966**		0.907		0.848	5 folds
			RF		0.762		-		0.786		0.759		0.938		**0.862**	
			Radial SVM		**0.841**		-		**0.862**		0.862		**0.959**		0.772	
				**Average**	**0.78**	**0.87**		**0.80**		**0.86**		**0.77**		**0.83**		**-**

RF: Random forest; LR: Logistic Regression; ACC: accuracy; AUC: area under the curve; PPV: positive predictive value; Sens: sensibility; Spec: specificity; CV: cross validation; LOOCV: Leave one out cross validation.

*ERP or bands that shows better results are highlighted in bold.

**It presents participants per group, followed by the distribution between males (M) and females (F), and the mean age and standard deviation.

SL approaches in PTSD psychotherapy are presented in Table VI. These studies only consider PTSD patients to assess therapy response with either TMS or tDCS. Only the analysis of EEG bands is reported, another BEAA techniques have not been explored. The unique feature used is the Power Spectral Density (PSD). The only ML model employed is SVM with a max accuracy of 0.817.

**TABLE VI:** Supervised Learning Approaches Used to Identify PTSD in Psychotherapy Contexts and Its Metrics

**Study/ Year**	**N° of Channels and bands** *	PTSD Participants**	**Therapy**	**Model**	**Feature**	**ACC**	**AUC**	**Sens**	**Spec**	**CV**
[70] 2021	8 **Alpha**, **Beta**, **Theta**, and **Delta** bands	47 pre-TBS/43 pos-TBS 39 M, 8 F 51.47±12.4	TMS with iTBDS	Lineal SVM with L1, and leave-two out CV	PSD	0.75	-	-	-	Not Mentioned
[73] 2022	62 Alpha, **Beta**, **Theta**, and Delta bands	48 23 M, 25 F 50.81±11.60	tDCS	SVM with Grid Search for C and γ	PSD	**0.817**	**0.93**	**0.77**	**0.87**	5 folds
					**Average**	**0.78**	**0.93**	**0.77**	**0.87**	-

ACC: accuracy; AUC: area under the curve; Sens: sensibility; Spec: specificity; CV: cross validation; TBS: theta burst stimulation.

*The bands that show better results are highlighted in bold.

**It presents participants per group, followed by the distribution between males (M) and females (F), and the mean age and standard deviation.

#### 4) Multimodal Learning Studies

Multimodal machine learning (MML) models are used in three diagnosis studies and are described in Table VII. Only EEG bands have been explored within the analysis of brain electrical activity. All multimodal learning models involved at least one additional data modality and performed an early data fusion by integrating these modalities into the EEG. [50] and [51] reported a single metric, F-Score and AUC, respectively. Only [55] reported accuracy, precision, recall, specificity, F-Score, MCC and AUC as metrics. Multimodal learning faces significant challenges due to data heterogeneity across formats and types (structured, unstructured, or semi-structured). Fusing these modalities entails difficulties in temporal synchronization, semantic alignment, computational complexity, and data quality [103], [104]. Studies [50] and [51] employ an elicitation tool to synchronously present stimuli and collect data from multiple sensors during interaction. In [50], early fusion is achieved via a bilinear factor model that projects both modalities into latent spaces, maximizing covariance. While [51] and [52] also implement early feature fusion, specific techniques are not detailed. All studies note increased computational demands with additional modalities, addressing this only through dimensionality reduction. Cross-validation was used to mitigate overfitting, with [51] uniquely incorporating repeated resampling.

**TABLE VII:** Multimodal Learning Approaches, Modalities, Fusion and Metrics Used in the Studies

**Study/ Year**	N° of Channels and bands*	PTSD/HC Participants**	**Model(s)**	**Type of fusion**	**Additional modalities**	**Feature(s)**	**Metric(s)**
[50] 2014	20 **Alpha**, **Beta**, **Gamma**, **Theta**, and **Delta** bands	Not mentioned	GNB with 20-folds CV	Early fusion	Speech	Spectral power of bands fused with audio and language descriptors-based features	F-score in GNV for: **EEG**: 0.61
							**Speech** and **EEG**: 0.61
			Linear SVM with 20-folds CV				F-score in SVM for: **EEG**: 0.62
							**Speech** and **EEG**: 0.68
[51] 2014	20 **Alpha**, **Beta**, **Gamma**, and **Theta** bands	30 Not mentioned	SVM with Grid Search, and CV with Bootstrapping	Early fusion	ECG, head motion signals, GSR and speech	Power-based spectral features fused with signal statistics, signal entropy and acoustic features	Only **EEG**: AUC = 0.62
							**All modalities**: AUC = 0.63
[55] 2020	24 **Alpha**, **Beta**, **Gamma**, **Theta**, and **Delta** bands	26/18 10M, 16F/10M, 8F 29.11±13.52	Random Forest with 5-folds CV	Early fusion	Free-hand sketches	Spectral power of bands fused with number of corners, and number and average length of strokes	Only **EEG**: ACC: 0.720, AUC: 0.798, F-score: 0.2
							**Sketches** + **EEG**: ACC: **0.993**, Prec: **0.993**, recall: **0.993**, Spec: **0.988**, MCC: **0.985**, AUC = **1.0**, F-Score: **0.993**
					**Average**		**AUC = 071 and F-Score = 0761**

GNB: Gaussian Naive Bayes; ACC: accuracy; Prec: precision, Spec: specificity; MCC: Matthews correlation coefficient; CV: cross validation.

*The bands that shows better results are highlighted in bold.

**It presents participants per group, followed by the distribution between males (M) and females (F), and the mean age and standard deviation.

## IV. Discussion

### A. Summary of Evidence

This scoping review provided a comprehensive examination of 73 studies utilizing electroencephalography (EEG) in the investigation of posttraumatic stress disorder (PTSD), particularly in diagnosis, differentiation, and therapeutical contexts. The analysis was performed across the entire EEG analysis pipeline, from a first stage that involves data acquisition and preprocessing to a second stage that involves statistical evaluation or machine learning analysis techniques.

The studies address various areas, from identifying EEG-based biomarkers to evaluating the efficacy of different interventions. Key findings include the utility of EEG in early detection and subtype differentiation using clusters. According to the map presented in Fig. 3, the trend in PTSD identification research leans towards a diagnostic context, primarily reporting experimental quantitative studies. There is also a significant focus on identifying PTSD evolution in psychotherapies, as these studies aimed to validate treatment effectiveness.

The review identified four main EEG-based brain activity analysis approaches: EEG bands, event-related potentials (ERPs), sleep characterization, and full-band EEG signal analysis. Overall, the analysis of EEG bands and ERP were found to be the most frequently used methods, particularly in diagnostic and differentiation settings, accounting for 51.85% and 33.3% of diagnostic studies, respectively. This preference for EEG band and ERP analyses in these contexts may be due to the ability of these techniques to capture specific changes in brain activity that are indicative of PTSD, thus allowing more accurate identification of the disorder. However, the limited use of techniques such as sleep characterization and full-band EEG signal analysis in contexts such as psychotherapy and pharmacotherapy may indicate a missed opportunity to explore their potential in PTSD therapy.

The analysis of the study population revealed a strong concentration in veterans and combatants (38.36% of studies). This concentration is understandable given that PTSD is more prevalent in these groups, but it also highlights a limitation in the population diversity of the studies, especially in the differentiation and pharmacotherapy contexts. In the PTSD differentiation, only veterans or combatants have been studied, which may limit the generalizability of the findings to other PTSD-affected populations. Furthermore, in 16 studies, the type of population is not explicitly mentioned, raising concerns about the transparency and reproducibility of the experiments. Moreover, the lack of clear information on demographic and clinical characteristics of participants, may difficult results comparison across studies and to perform studies on data and model bias.

Overall, the identified open datasets demonstrate the diversity of sample sizes, EEG configurations, and participant characteristics in different mental health research contexts. Also, small sample sizes (between 12 and 30 participants) are evident.

During the EEG recording and feature representation phase, only traditional methods—such as univariate filters, wavelet decomposition, and conventional feature extraction techniques—have been implemented. Advanced approaches, such as adaptive multivariate decomposition, which have shown promising results in diagnostic and intervention contexts, remain unexplored in PTSD analysis.

Two research approaches were identified for building computational EEG-based models for the identification of PTSD. The first involved comparing EEG features, using conventional statistical measures, to evaluate signal variations among PSTD and healthy subjects. The second approach is using machine learning techniques (supervised, unsupervised, and multimodal). In general, due to the limitations on the availability of fully open raw EEG recordings, annotated and validated with PTSD questionaries, it has not been possible the development of reliable computational models to identify patterns in the EEG signal, to support PSTD diagnosis, differentiation and therapy.

The supervised learning approach was reported in diagnostic, differentiation, and psychotherapy contexts. SVM is the most widely used method in the three contexts, being the one with the best results. Along with this method, deep learning methods have also been explored in PTSD diagnosis, such as EEGNet (0.999), which have managed to improve the accuracy results obtained with SVM (0.997). In differentiation, the best results were given by SVM and C5.0, with 0.841 accuracy for both models. There is no evidence that deep learning methods have been explored in differentiation. In psychotherapy, SVM is the only method explored, and the mean accuracy is 0.78. In these supervised learning approaches, sleep characterization has not been explored and may be an important aspect to consider in future research. ERPs have also been little explored, only ERP P300 has been studied and only in diagnostic and differentiation contexts. The results obtained with this ERP are promising, so it might be worth exploring other ERPs such as (P1, P2, LPP, VPP, among others) and apply them to the context of psychotherapy. Finally, several signal features have been studied, which can be grouped into time, frequency, statistics, and morphological ones. Particularly in differentiation, the features with the best results are those related to amplitude and power, while in psychotherapy power is relevant.

In the unsupervised machine learning approach, only diagnostic and differentiation contexts have been explored. In PTSD diagnosis, the models prove useful applications for grouping clusters into PTSD subtypes or symptomatic groups. In general, only the use of the Calinski-Harabasz index as a measure of dispersion between clusters or between data is evident. Therefore, more research is necessary in unsupervised ML, however, this is not feasible without large scale datasets as they are found in other clinical areas (neuroimaging, radiography, and genomic analysis)

The multimodal machine learning approach has only been applied in the context of PTSD diagnosis. In this scenario, only EEG bands have been considered as a method of analyzing EEG-based brain electrical activity. The studies analyzed demonstrate, however, that incorporating multiple data modalities does increase the performance of the models.

Nevertheless, the above findings open the door for continuing research on MML. This type of learning is characterized by integrating multiple modalities related to physiology, movement, speech, drawing and text. Here, only classical methods such as SVM and Random Forest have been explored. No other classical methods or deep learning related methods have been deployed. Particularly, considering that interviews, audio, videos, and free text are formats frequently used as diagnostic instruments, multimodal LLM (Large Language Models) have the potential to contribute to the advance of this research field. Again, a reliable multimodal dataset is a pre-requisite.

### B. Challenges and Limitations of Current Computational Methods for EEG-Based PTSD Analysis

Several studies in the field of PTSD rely on small sample sizes, primarily consisting of combatants and veterans, limiting the generalizability and robustness of their findings. Additionally, the absence of detailed demographic and clinical information in certain studies hinders inter-study comparisons and increases the risk of bias in data and modeling. Furthermore, the lack of standardized data collection and analysis protocols complicates cross-study comparisons, impeding reproducibility and the translation of research findings into clinical practice.

Univariate EEG-based approaches, due to their limited time-frequency resolution, are insufficient to capture the non-linear, non-stationary, and complex nature of EEG signals. In contrast, advanced techniques such as adaptive, iterative, and multivariate methods have demonstrated promising results in diagnosis and intervention. Their application in PTSD research could significantly contribute to the identification of the disorder. Moreover, the high dimensionality and noise inherent in EEG data necessitate sophisticated feature extraction techniques to ensure meaningful representations while preserving interpretability.

Current research predominantly focuses on conventional EEG features, neglecting the potential of novel feature extraction methods, such as multiple iterative decomposition, and the impact of feature engineering strategies. Traditional EEG analysis techniques, including band analysis, event-related potentials, sleep characterization, and whole-EEG analysis, have been widely employed. However, key areas like sleep characterization and full-band EEG analysis remain underutilized in contexts such as differentiating PTSD from other disorders or evaluating therapeutic interventions. While deep learning has shown promise in diagnosis, its application in other contexts and within other machine learning frameworks remains largely unexplored. This gap hinders a comprehensive understanding of the diagnostic and interventional potential of EEG analysis.

Multimodal approaches, integrating EEG with other modalities like audio or physiological signals, present additional challenges, including data synchronization, semantic alignment, and computational overhead. These challenges are further compounded by individual variability influenced by factors such as age, health status, and trauma history. To address these complexities, innovative computational frameworks are required to harmonize multimodal data while balancing complexity, interpretability, and diagnostic accuracy. Expanding the exploration of underutilized EEG analysis domains is essential to advance the field of PTSD research.

### C. Limitations of the Review

This scoping review only considered EEG neuroimaging technique due to its low cost, portability, and high temporal resolution. However, studies involving techniques such as fMRI or MEG, which offer better spatial resolution, might improve the performance of evaluated models, and could be useful for better understanding brain connectivity and obtaining biomarkers. Additionally, the heterogeneity in diagnostic criteria and study populations across included studies may have influenced the interpretation of findings. Moreover, the predominance of cross-sectional studies limits the ability to reflect the longitudinal dynamics in PTSD, indicating a potential gap in the literature that warrants further exploration.

## V. Conclusion

The deep analysis of the 73 studies included in this review demonstrated the potential application of EEG in supporting diagnostic, differentiation and therapeutic processes of PTSD. In the studies, distinct types of population PTSD have been considered, but mostly veterans or combatants. Evidence shows that PTSD identification is possible from the analysis of brain activity measured with EEG Bands, ERP, sleep characterization or the complete EEG signal. The review highlights a predominant focus on the use of EEG and ERP band analysis for the diagnosis and differentiation of PTSD. Alpha, beta y theta bands provide better identification. In contrast, the gamma band is one of the bands that did not provide much information at the time of identification. Regarding sleep characterization, studies show that the N1 and N2 stages of NREM sleep are more suitable for identification of the disorder. Finally, better performance results have been obtained when using the bands over the whole EEG data. Nevertheless, one of the biggest limitations in analyzing and characterizing EEG-based brain activity for PTSD, is the limited availability of EEG datasets with raw data recordings.

Regarding the use of ML models for PTSD, supervised machine learning models have shown better performance, especially in PTSD diagnosis, with SVM as the most recurring and effective method. However, there is a lack of applied studies in psychotherapy considering other ML methods, population, and EEG-based brain activity analysis methods, limiting the comparison between different techniques and patient groups. Unsupervised learning approaches in the context of PTSD identification are limited to diagnosis and differentiation contexts. Studies demonstrated that unsupervised models are useful, k-means particularly for the identification of subtypes within patient groups and AHC for differentiating between different disorders.

In multimodal learning, SVM and Random Forest have shown improved classifier performance. However, such improvements are marginal compared to using EEG data alone. In this sense, the improvement does not depend on the number of data modalities to be integrated, but rather on how informative the modality is. Furthermore, other types of data fusion techniques have not been explored.

## Supplementary Materials

Section 1 details the methods used to include and characterize the studies. Section 2 complements the results presented in this main manuscript, using complementary tables and figures.

Supplementary Materials

## Conflict of Interest

The authors declare no conflict of interest.

## Author Contribution

Each author contributed their expertise in their respective fields of research, from collecting studies, reviewing and extracting data to constructing the manuscript.

José A. Salazar-Castro drafted the manuscript. Diego M. López and Diego H. Peluffo-Ordóñez reviewed and edited the manuscript.

All authors read and thoroughly revised the final version of the manuscript.
