# Supplementary material for: Advances in Electroencephalography for Post-Traumatic Stress Disorder Identification: A Scoping Review
Source: IEEE Open J Eng Med Biol. 2025 Feb 5;6:332–44. doi: 10.1109/OJEMB.2025.3538498 (PMC12250877; doi:10.1109/OJEMB.2025.3538498)
Supplement: Supplementary Materials [file supp1-3538498.pdf]

Supplementary Materials

Advances in Electroencephalography for Post-traumatic Stress Disorder Identification: A Scoping Review

Salazar-Castro Jose A., Peluffo-Ordóñez Diego H., and López Diego M.

I. Methods

A. Protocol and Registration

In formulating the research question that underpins this review, we employed the PICO (Population-Intervention-Comparison-Outcome) framework. The target population comprises individuals exposed to traumatic events, with the intervention being the measurement of brain activity using EEG. For comparison, we assess computational techniques applied to EEG signals in the identification of PTSD. The outcome focuses on the efficacy of EEG signals in PTSD detection, evaluated through statistical measures and machine learning performance metrics. The central research question is: How can computational processing of EEG signals be used to develop an objective and accurate method that allows the identification of PTSD, based on the analysis, characterization and/or classification of EEG signals?

The detailed protocol can be consulted in the following link: https://docs.google.com/document/d/1GTQWKrmIaFHmyBbvLJyqStzacAvEthgG/edit?usp=sharing&ouid=111741533406664883496&rtpof=true&sd=true.

B. Selection of Sources of Evidence

During the initial selection phase, 170 articles were evaluated, excluding duplicates and one retracted article. The review was conducted by analyzing titles and abstracts, applying predefined eligibility criteria. Each article was assessed considering the EEG signal processing and characterization, target population and its diagnostic, as well as the evaluation techniques or procedures used in the identification of PTSD. A total of 76 abstracts were excluded for not meeting all inclusion criteria. In cases of insufficient or ambiguous data, a consensus was reached to review the full text to make a definitive decision on inclusion or exclusion.

C. Data Charting Process

The data extraction template implemented is presented in S-Table I.

D. Data Items

To ensure systematic and consistent data extraction, the tabulation template described in Supplementary Table I (S-Table I) was employed throughout the review. Data extraction primarily relied on information from titles and abstracts; however, when data were unclear or incomplete, the entire document was reviewed to obtain the necessary information. The template covered crucial elements such as study type, mental health-related application context, EEG-based electrical activity analysis, population of the study and its characteristics, type of comparison group (in the case it was considered), techniques, methods or models used in the identification of PTSD and the metrics used in the case of machine learning approaches. In the application context in mental health, we consider two diagnostic approaches: a) the diagnosis compared to a control group of healthy patients and b) the differential diagnosis from a control group of patients with other disorders that may or may not include a group of healthy patients (consider such as differentiation). Two therapeutic interventions were also examined: psychotherapy and pharmacotherapy. In both cases, control groups were compared to PTSD patients either untreated or receiving traditional treatment (TAU). The EEG-based Brain Electrical Activity Analysis (BEAA) was categorized into four dimensions: EEG band(s), Event Related Potential(s) (ERP), sleep characterization or full-band EEG. The sources of evidence were characterized through bibliometric mapping and synthesized via a scoping review.

E. Synthesis of results

The included studies were grouped considering the type of study and the mental health application context (diagnosis, differentiation, or therapy). Initially, a bibliometric analysis mapping is carried out to provide an overview of publications by year, most relevant authors, and the number of studies interrelating the type and context. Subsequently, in accordance with the objective of this review, only primary studies are considered. For the primary studies, the trends of each study were analyzed along with the characteristics, population, techniques, control groups used, mental health approach, and metrics used in the identification of PTSD, as well as the general findings presented for the studies.

II. Results

A. Selection of Sources of Evidence

A total of 316 duplicate records were removed from the initial 881, leaving 565 documents to be screened based on title and abstract. During this process, one study was excluded due to an author retraction, and 394 documents were filtered out. Subsequently, 170 documents were selected for a more detailed assessment and subsequent data extraction.

S-TABLE I

DESCRIPTION OF THE DATA TABULATION TEMPLATE FOR EACH ELIGIBLE EVIDENCE SOURCE

Item	Description	Example 1 – with machine learning model	Example 2 – with feature evaluation
**ID**	Study number	1	48
**Title**	Full title of the document for easy reference and location	A Novel Neurophysiological Feature Based on Quantifying EEG Data for Separating of Patients in Psychiatric Disorders with Comorbidities	Neurobiological Features and Response to Eye Movement Desensitization and Reprocessing Treatment of Posttraumatic Stress Disorder in Patients with Breast Cancer
**Autor(es)**	Authors list	Shim, M; Lee, S.-H.; Hwang, H.-J.	Carleto et al.
**Year**	Year of publication	2022	2019
**Abstract analysis**	Presents the most relevant findings of the summary that are important for this study.	The study considers the similarity between PTSD and MDD at the symptomatic level, which involves difficulty in the diagnosis of both diseases. They propose a feature extraction method to determine a CAD system to differentiate these two groups. For this, they use ERP and characterize by averaging the waveforms of the P300 event in 30 EPT patients and 30 MDD patients. They classify with SVM with leaving 1 out cross-validation and obtain an accuracy of 73.33% considering a mean characteristic based on the amplitude.	They evaluated the efficacy of eye movement desensitization and reprocessing (EMDR) therapy compared to treatment as usual (TAU) using EEG. EEG was used to corroborate the findings that patients treated with EMDR no longer met the criteria for PTSD while those treated with TAU maintained the diagnosis. EEG demonstrated significant differences in the delta and theta bands in the left angular and right fusiform gyri only in the EMDR group.
**Type of study**	Classifies each study according to its nature and design such as primary (P) or secondary (S) studies. The categories considered include:<br>Experimental studies (P*)<br>Case reports (P*)<br>Theoretical studies (S*)<br>Reviews or meta-analysis (S*)	Experimental study	Experimental Study
**Application context in Mental health**	The mental health approach considers the purpose of EEG processing in the PTSD identification and is determined between four categories:<br>Diagnosis (vs health control group)<br>Differentiation (vs other mental disorders)<br>Pharmacotherapy<br>Psychotherapy	Differentiation (Differentiate PTSD from MDD)	Psychotherapy
**Type of Brain activity Analysis**	It involves the approach carried out in the analysis of the EEG signal, whether it is analysis of EEG bands, sleep characterization, EEG full-band or ERP analysis	ERP Analysis	EEG bands Analysis
**Type of EEG band or ERP***	This item establishes the type(s) of bands and/or ERP, and the main characteristic used in the study. The best results are highlighted in bold	Amplitude of the **P300** ERP	Power Spectral Density (PSD) of d**elta**, **theta**, alfa and beta
**Study Population**	Identifies the type of population involved in the study, such as veterans/combatants, victims of natural disasters, victims of accidents, not mentioned, etc.	Not mentioned	Other type of victims
**Comparison Group**	The type of comparison group is detailed if it is with a control group of healthy patients, only patients with PTSD, or patients with other disorders.	30 with PTSD and 30 with MDD, based on DSM-IV<br>15 male, 15 female and 9 male, 21 female<br>42.77 ± 11.16 and 44.60 ± 10.08 years old	30 PTSD based on DSM-V, 15 with TAU and 15 EMDR<br>All females<br>48.40 ± 9.42 and 55.47 ±7.64 years old
**Type of EEG model/technique analysis**	Describes the statistical techniques used to compare the features or machine learning models used along with their configurations.	Linear SVM classifier with LOOCV method	Pearson correlation<br>t-test<br>ANOVA<br>Multivariate linear regression
**Model evaluation metrics**	If the EEG analysis is based on machine learning models, the metrics and the best values are reported.	Accuracy: 73,33%<br>sensitivity: 90%<br>specificity: 56.67 %	NA

*The band(s) or ERP that shows better results are highlighted in bold.

The studies were categorized into primary and secondary. n the primary group, 58 articles were excluded for the following reasons: 14 did not present conclusive EEG-based solutions, 18 did not involve patients diagnosed with PTSD, 15 relied on other neuroimaging techniques, 1 involved prior treatment or medication use before EEG acquisition, and 10 did not focus on PTSD identification using EEG. In the secondary group, 18 articles were excluded: 8 addressed other neuroimaging or physiological signals for PTSD identification, and 10 did not provide information on research gaps, challenges, limitations, or solutions related to PTSD identification using EEG. Finally, 94 documents were considered eligible for this review, 73 primaries and 21 secondaries studies. Fig. 1 presents a flowchart illustrating the study selection process visually and is presented in the main manuscript.

B. *Characteristics of Sources of Evidence*

This subsection presents the supplementary bibliometric analysis, which examines the number of studies published per year, the most prominent authors in the field, and a co-occurrence analysis.

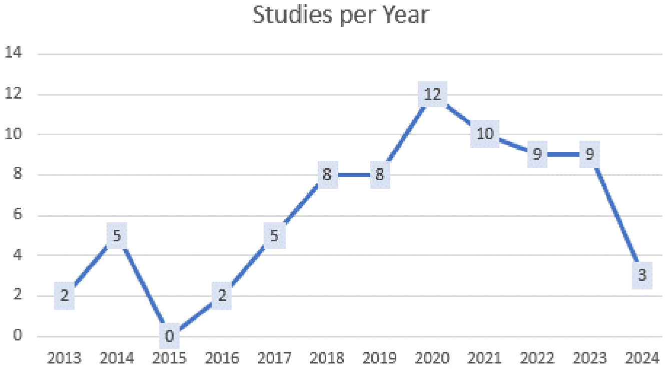

S-Fig. 1. Scientific production of chosen documents distributed by year. A growing trend of studies starts in 2017 onwards and a peak in 2020 and as of 2021 has been maintained in 9 studies under our inclusion criteria.

Initially, a growing trend was observed in publications focused on PTSD identification through EEG signals. Starting in 2018, there was a marked increase in annual output, with at least eight publications per year. The year with the highest output was 2020, with a peak of 12 publications. Since 2021, the number of publications has stabilized at nine per year, based on our inclusion criteria. This trend is illustrated in S-Fig. 1.

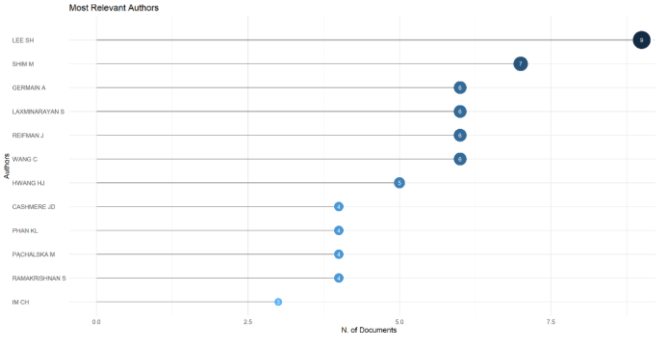

S-Fig. 2. Most prolific authors in the field of PTSD identification on PTSD diagnosis, differentiation, and therapy using EEG, considering the number of published documents between experimental studies and case reports.

S-Fig. 2 presents the most relevant authors in the field, highlighting Lee S. H. as the author with the most publications (n = 9). This author has made significant contributions in PTSD diagnosis, differentiation, and psychotherapy. In terms of case studies, Pąchalska M. is the author with the most contributions in this type of research in the context of PTSD diagnosis (n = 1) and treatment (n =3).

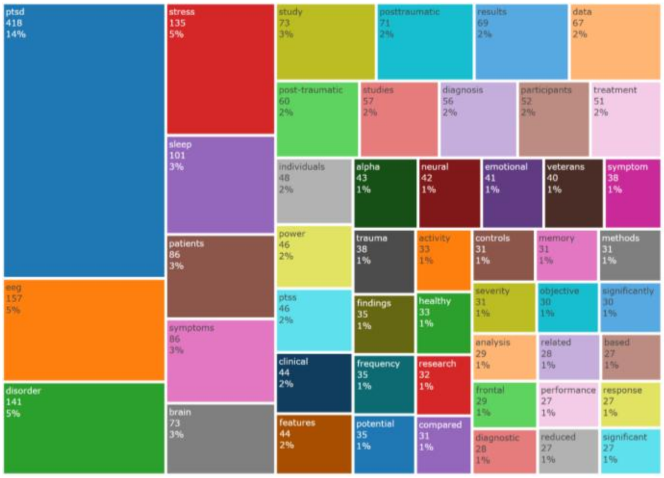

S-Fig. 3. A word cloud depicting the most frequently used terms across the analyzed abstracts of the documents.

S-Fig. 3 presents a word cloud showcasing the most frequent terms in the analyzed documents. Notable keywords include synonyms of PTSD, EEG-related terms, signal acquisition conditions, and machine learning techniques such as Support Vector Machines (SVM). S-Fig. 4 represents a topic co-occurrence network, showing how topics integrate into thematic clusters. Three research niches are identified: PTSD, computational techniques, and EEG features.

C. *Results of Individual Sources of Evidence*

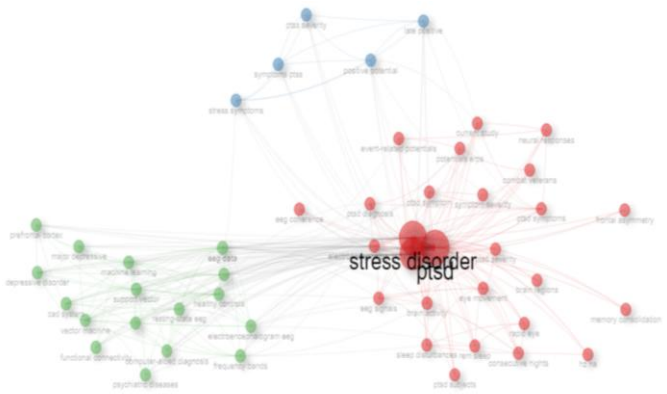

S-Fig. 4. Topic co-occurrence network where we can find three key groups regarding PTSD diagnosis

Due to the number of sources included in this scoping review, relevant data from each source is provided in a supplementary file: https://drive.google.com/drive/folders/14v40_H1kXjE1QoXInfIxOyKUo8JVfecG?usp=sharing

D. *Synthesis of Results*

73 primary studies were considered for the synthesis of results, with 69 experimental studies [2], [7], [11], [19], [27], [29-71], [73-75], [77-95] and 4 case reports [72], [76], [83], [96]. Some of the remaining 21 secondary studies, 5 theoretical

[5], [14], [18], [99], [100] and 16 reviews [4], [16], [20-26], [101-102], were used to expand the discussion and strengthen the argument in the results section.

1) *Type of EEG-based Brain electrical activity Analysis*

We complement the analysis of the brain electrical activity analysis (BEAA) presented in Table I of the main text of the manuscript.

a) *Full-band EEG*

Three studies focusing on the full-band EEG analysis include EEG signal characterization, image rendering techniques or data augmentation, as presented in S-Table II. All studies perform a segmentation stage, which proves to be very useful considering the non-stationarity of EEG data [32]. These studies involve data extraction by processing the complete EEG signal, without the need to extract frequency bands or ERPs. In [32], a Hurst analysis with a scaled range is used to identify EEG signals that differentiate between healthy controls and subjects with PTSD. They show that the Hurst Exponent value is lower in the F3 channel in PTSD patients. In [33], the technique measures brain activity directly with EEG to obtain a representation in time-frequency maps using the Continuous Wavelet Transform (CWT). Thus, these maps are converted into RGB images that are the inputs to a pre-trained CNN model. [34] and [35] perform an analysis of the full-band EEG, and they compare it with the typical EEG bands extracted from the full-band. Finally, in [36] the full-band EEG of participants with and without PTSD is used to determine the effects of different cross-validation (CV) strategies on classifier performance within a data augmentation context.

S-TABLE II
SEGMENTS SIZE AND METHOD USED IN FULL-BAND EEG ANALYSIS

Study Year	Channels and Participants*	Segments window (s)	EEG processing method
[32] 2018	31 channels<br>6 PTSD/6 HC<br>All males	131	Dynamical Hurst Analysis
[33] 2022	16<br>15 PTSD/30 HC<br>All males	1	Continuous Wavelet Transform
[36] 2023	64<br>77 PTSD/58 HC<br>28M, 49F/30M, 28F	5, 10, 15, 20 and 60	CNN to extract features

*It presents the number of participants per group, followed by the distribution between males (M) and females (F), and the mean age and standard deviation.

b) *Sleep characterization*

In these studies, sleep characterization is considered in the analysis of sleep stages, that could be Awake (W), Rapid Eye Movement [REM], or non-REM [NREM] in stages N1, N2, and N3. In [37], the authors present a study that considers alterations in the EEG during sleep stages, but it is not a specific characterization of the stage, it is an extraction of EEG bands to analyze those. In same way, in [38] during the REM and NREM sleep the EEG bands were extracted and analyzed but not throw a direct characterization of sleep stages. A similar process is realized in [39], [40] and [41]. For this reason, those studies are grouped in EEG bands analysis. S-Table III shows the stage of sleep considered in the sleep characterization studies. In [42], 10 sleep-related outcome variables were deployed and specifically two, number of spontaneous arousals (NSA) and slow-wave sleep (SWS) percentage in the slow spindle (SS), were extracted from the EEG. [43] consider spectral power in the different stages of sleep, considering in addition the awake state. [44] consider analyze the fast spindle (FS) and SS, and [45] only FS. The slow spindle (SS) and fast spindle (FS) are sleep-related bands in the 10-13Hz and 13-16Hz, respectively. S-Table IV presents the features analyzed from the SS and FS.

S-TABLE III
SLEEP STAGES CONSIDERED IN PTSD DIAGNOSIS BASED ON SLEEP CHARACTERIZATION

Study Year	Channels and Participants*	W	REM	NREM				
				N1	N2		N3	
					SS	FS	SS	FS
[42] 2014	16 channels<br>16 PTSD/29 HC<br>All females	-	✓	-	✓	-	-	-
[43] 2019	6 channels<br>38 PTSD/38 HC<br>Not mentioned	✓	✓	✓	✓	✓	-	-
[44] 2020	64 channels<br>37 PTSD/46 HC<br>30M, 7F/45M, 1F	-	-	-	-	-	✓	✓
[45] 2020	64 channels<br>37 PTSD/46 HC<br>30M, 7F/45M, 1F	-	✓	-	✓	✓	✓	-

W: Awake; REM: rapid eye movement; NREM: non-rapid eye movement; SWS, slow-wave sleep; TE, trauma-exposed non-PTSD; SS: slow spindle; FS: fast spindle.
*M: male(s); F: female(s)

S-TABLE IV
FEATURES DEPLOYED IN EACH SLEEP SPINDLE IN PTSD DIAGNOSIS

Study	SS	FS	A	T	OF	D	PLV	MPD
[44]	✓	✓	✓	✓	✓	✓	-	-
[45]	✓	✓	-	-	-	-	✓	✓

A: amplitude; T: duration; OF: oscillatory frequency; d: density, PLV: phase-locking value; MPD: mean phase difference.

c) *EEG Bands*

This subsection discusses primary studies focusing on the diagnosis (vs. healthy patients), differentiation (vs. patients with other disorders), and PTSD psychotherapy through the characterization of EEG bands. In these studies, EEG bands are extracted from the full-band EEG in specifics range of frequency. The approximate frequency ranges include delta (0.5–4 Hz), theta (4–7 Hz), alpha (8–12 Hz), beta (13–30 Hz), and gamma (31–60 Hz). Certain studies extend the analysis to other frequency ranges: for instance, [37] includes a sigma band (12–16 Hz) and slow oscillations in the 0.5–1 Hz range, while [34] utilizes a full-band EEG from 0.1 to 100 Hz, and [35] focuses on the 4–50 Hz range. Specific frequency bands also appear, such as the Slow-Wave Frequency Band (SWA) in the 1–4.5 Hz range in [41], and broadband in the 1–30 Hz range in [46] and 1–40 Hz for [47]. Additionally, [48] defines a high-frequency band from 23–36 Hz.

S-Table V presents the distribution of these bands across studies focusing on diagnosis, differentiation, and psychotherapy. There is only one pharmacotherapy study [31] that explores the use of the gamma band, along with P50 and ASSR ERP, to assess the pharmacological effects in PTSD patients.

S-TABLE V
EEG BANDS EXPLORED IN INCLUDED STUDIES CONSIDERING THE DIAGNOSIS, DIFFERENTIATION OR PSYCHOTHERAPY OF PTSD

Context	Study/ Year	Participants***	Alpha	Beta	Sigma	Gamma	Theta	Delta	Specific*	Full**
Diagnostic	[49] 2013	219 PTSD/22 HC<br>152 M, 89 F<br>52.9±7.8	✓	✓	-	-	✓	✓	-	-
	[50] 2014	Not mentioned	✓	✓	-	✓	✓	✓	-	-
	[51] 2014	30<br>Not mentioned	✓	✓	-	✓	✓	-	-	-
	[52] 2017	See in Table VI	✓	-	-	-	-	-	-	-
	[53] 2018	147 PTSD<br>Not mentioned<br>27.8±7.9	✓	✓	-	-	✓	✓	-	-
	[54] 2018	24 PTSD/30 HC<br>6 M, 18F/6 M, 24 F<br>30.7±9.9/28.9±8.7	✓	-	-	-	-	-	-	-
	[2] 2020	29 PTSD/13 HC<br>Not mentioned<br>32.24±7.38/32.00±5.70	-	-	-	-	✓	-	-	-
	[7] 2020	See in Table VI	✓	✓	-	✓	✓	-	-	-
	[29] 2020	See in Table III	✓	✓	-	✓	✓	✓	-	-
	[34] 2020	15 PTSD/ 30 HC<br>All males<br>52.82±4.28/50.87±3.87	✓	✓	-	✓	✓	✓	-	✓
	[35] 2020	See in Table III	✓	✓	-	✓	✓	✓	-	✓
	[37] 2020	See in Table III	✓	✓	✓	✓	✓	✓	✓	-
	[38] 2020	Same that [37], see in Table III	-	✓	✓	✓	✓	✓	-	-
	[39] 2020	Same that [37], see in Table III	✓	✓	✓	✓	✓	✓	✓	-
	[55] 2020	26/18<br>10 M, 16 F/10 M, 8 F<br>29.11±13.52	✓	✓	-	✓	✓	✓	-	-
	[27] 2021	Same that [7], see in Table VI	✓	✓	-	✓	✓	-	-	-
	[40] 2021	37/47<br>30 M, 7 F / 46 M, 1 F<br>31.3±5.0/32.6±5.7	✓	✓	✓	-	✓	✓	-	-
	[41] 2021	10/10HC<br>Not mentioned<br>14.52±0.95/14.67±0.94	-	✓	✓	✓	-	-	✓	-
	[56] 2021	34 HS PTSD/26 LS PTSD<br>11 M, 23 F/16 M, 10 F<br>16.29±0.46/16.12±0.33	✓	✓	-	-	✓	✓	-	-
	[57] 2021	See in Table III	-	✓	-	-	✓	✓	-	-
	[19] 2022	See in Table III	✓	✓	-	✓	✓	✓	-	-
	[46] 2022	See in Table III	✓	✓	-	-	✓	✓	✓	-
	[58] 2022	See in Table III	✓	✓	-	✓	✓	✓	-	-
	[59] 2023	See in Table III	✓	✓	-	-	-	-	-	-
	[60] 2023	See in Table III	✓	✓	-	✓	✓	✓	-	-
	[61] 2023	30 PTSD<br>12 M, 18 F<br>37.70±10.76	✓	✓	-	✓	✓	✓	-	-
	[62] 2023	See in Table III	✓	✓	-	✓	✓	✓	-	-
	[63] 2024	See in S-Table VIII	✓	✓	-	-	✓	✓	-	-
**Total Per Band in Diagnostic**			**24**	**25**	**5**	**17**	**24**	**20**	**4**	**2**

S-TABLE V-CONTINUATION
EEG BANDS EXPLORED IN INCLUDED STUDIES CONSIDERING THE DIAGNOSIS, DIFFERENTIATION OR PSYCHOTHERAPY OF PTSD

Context	Study/ Year	Participants***	Alpha	Beta	Sigma	Gamma	Theta	Delta	Specific*	Full**
**Differentiation**	[64] 2016	See in S-Table VIII	✓	✓	-	✓	✓	✓	-	-
	[65] 2018	See in S-Table VIII	✓	✓	-	-	✓	✓	-	-
	[66] 2021	See in S-Table VIII	✓	✓	-	✓	✓	✓	-	-
	[67] 2023	See in S-Table VIII	✓	✓	-	-	✓	✓	-	-
	[63] 2024	See in S-Table VIII	✓	✓	-	-	✓	✓	-	-
**Total Per Band in Differentiation**			**5**	**5**	**0**	**2**	**5**	**5**	**0**	**0**
**Psychotherapy**	[48] 2017	19 PTSD<br>Under 11	-	-	-	-	-	-	✓	-
	[68] 2017	21 PTSD<br>3 M, 18 F<br>39.9±13.7	✓	-	-	-	-	-	-	-
	[69] 2019	15 TAU/ 15 EMDR<br>All females<br>48.40±9.42/55.47±7.64	✓	✓	-	-	✓	✓	-	-
	[70] 2021	See in Table V	✓	✓	-	-	✓	✓		-
	[71] 2022	29 PTSD<br>All female<br>53.72 ± 6.09	✓	✓	-	✓	-	-	-	-
	[72] 2022	1 PTSD<br>Female<br>67 years old	✓	-	-	-	-	-	-	-
	[73] 2022	See in Table V	✓	✓	-	-	✓	✓	-	-
	[47] 2023	38 PTSD/32 HC<br>11 M, 27 F/ 10 M, 22F<br>39.20±12.08/42.40±10.7	✓	✓	-	-	✓	✓	-	-
	[74] 2023	300 PTSD<br>150 M, 150 F<br>Rango of 34 to 75	✓	-	-	-	-	-	-	-
	[75] 2024	Same that [69]	✓	✓	-	-	✓	✓	-	-
**Total Per Band in Psychotherapy**			**9**	**6**	**0**	**1**	**5**	**5**	**1**	**0**
**Pharmacotherapy**	[31] 2019	23 PTSD<br>Male and Female<br>Range of 21 to 65	-	-	-	✓	-	-	-	-
**Total Per Band in Pharmacotherapy**			**0**	**0**	**0**	**1**	**0**	**0**	**0**	**0**

*Some studies include a specific range of band that could be different between them.

**The full band is characterized and compared with another band extracted from the full band. The studies that realize this process were included into the EEG-band analysis but not into the full-band analysis.

***M: male(s); F: female(s)

According to S-Table V, the most used EEG bands in PTSD identification are Alpha (n = 38), Beta (n = 36) and Theta (n = 34). The least explored bands are sigma (n = 5) and the full band (n =2) and are used only in diagnosis contexts. The specifics bands (n = 5) are used in almost two contexts. Considering the pharmacological study, gamma is the unique band explored in the four contexts. However, studies indicate that the alpha band yields better results for diagnosis and psychotherapy, while beta excels in differentiation. We have highlighted in bold the bands that, according to these studies, exhibit the greatest difference between the group with the disorder (or with probable PTSD) and the control group(s).

These findings may be influenced by the characterization processes, PTSD identification techniques, or models employed. As shown in Table III of the main manuscript, all bands in [46] produced significant results for classifying PTSD patients compared to healthy individuals. However, in [7] and [29], the theta band showed the strongest results. All three studies used a linear SVM classifier, but differed in participant numbers, features, and model-fitting processes.

d) ERP

This subsection also presents the analysis in diagnosis, differentiation and therapy of PTSD, but considering the characterization of Event Related Potentials (ERP). ERPs are defined by specific waveforms that occur at predetermined time intervals following sensory, cognitive, or motor events. The studies included stimuli such as images, emotional words, auditory and olfactory signals, audiovisual combinations, and tasks like Go/No-Go, flanker tasks, and working memory evaluations. ERPs that have been studied in more than one context showing differences in the identification of PTSD are P300, used in diagnosis and differentiation; Late Positive Potential (LPP) and P3, used in diagnosis and psychotherapy;

and Auditory Stady State (ASS), used in diagnosis and pharmacotherapy. An important clarification is that P100 and P1, P200 and P2, P300 and P3, N100 and N1 are considered similar because they occur at the same time instants. Here we have respected the notation assigned by the authors. If we consider P300 and P3 as the same potential, then this potential would be the only one that has been studied in three contexts. The detailed ERP analysis for each context is available in S-Table VI.

Furthermore, [29] used P300 to validate the correct calculation of source signals, while frequency bands were employed as the primary technique to classify between PTSD and healthy patients. Another consideration arises with [30]. This study focuses on both diagnosis (including a healthy control) and differentiation (from a major depressive disorder (MDD) group). It has been included in both diagnosis and differentiation using ERP. The psychotherapy case study in [76] used both ERP and EEG bands analysis, but as the EEG bands

S-TABLE VI
DISTRIBUTION OF EVENT-RELATED POTENTIALS (ERPS) ACROSS STUDIES FOCUSED ON PTSD DIAGNOSIS, DIFFERENTIATION, AND THERAPY

Context	Study/ Year	Participants*	ERP
Diagnostic	[77] 2013	19 PTSD/14 HC<br>All males<br>29.95 (8.03) / 34.71 (8.80)	**LPP**, **VPP**
	[78] 2014	42 PTSD<br>5 M, 38 F<br>20(1.96)	**LPP**
	[79] 2014	18 PTSD/16 HC<br>17 M, 1 F/15 M, 1 F<br>33.5 ± 7.2 / 33.5 ± 7.2	**ONE**
	[80] 2016	28 PTSD/30 HC<br>10 M, 18 F/16 M, 14 F<br>51.39 (5.18) /49.63 (6.38)	**CNV**
	[81] 2017	51 PTSD<br>11 M, 40 F<br>28.10 (9.62)	**ENR**, **REWP**
	[82] 2017	21 HS PTSD /21 LS PTSD<br>Not mentioned<br>29 (6.9)	N100, N170, N200, **P100**, P300, **VPP**
	[83] 2018	24 PTSD/24 HC<br>All males<br>36.83 (8.26) / 26.92 (9.32)	**LPP**
	[84] 2018	1 PTSD, 23 HC<br>1 male<br>44 years	**P3**
	[30] 2019	See in S-Table VIII	**P300**
	[85] 2019	77 PTSD<br>28 M, 49 F<br>39.9±12.5	**N170**
	[86] 2019	21 HS PTSD/21 LS PTSD/ 17 HC<br>5 M, 16 F/9 M, 12 F/6 M, 11 F<br>25.60±4.58/ 24.47±2.41/23.85±1.77	**N1**, **P3**
	[87] 2019	27 HS PTSD/25 LS PTSD/18 HC<br>17 M, 10 F/16 M, 9 F/8 M, 10 F<br>24.52±5.21/23.24±6.11/21.72±4.98	**N170**, NSW, **VPP**
	[29] 2020	See in Table III	**P300**
	[88] 2020	80 PTSD<br>67 M, 14 F<br>33.72±6.47	**LPP**
	[89] 2021	52 PTSD<br>44 M, 8 F<br>51.3±7.0	**LPP**
	[90] 2021	35 PTSD/19 HC<br>29 M, 6 F/19 M, 0 F<br>33.3±8.6/33.4±9.7	N1, **P2**, P3
	[91] 2022	14 HS PTSD/11 LS PTSD/23 HC<br>16 M, 41 F<br>21.6±2.8	**LPP**
	[11] 2023	17 PTSD/32 CPTSD/32 HC<br>1 M, 16F/5 M, 27 F/10 M, 22 F<br>17.65±2.06/17.72±2.53/16.59±1.04	EPN, LPP. P100
	[92] 2024	25 PTSD/25 HC<br>20 M, 5 F/22 M, 3 F<br>42.13±16.11//40.25±13.64	**ASS**
**Total different ERP explored in diagnosis**			**17**

S-TABLE VI-CONTINUATION
DISTRIBUTION OF EVENT-RELATED POTENTIALS (ERPS) ACROSS STUDIES FOCUSED ON PTSD DIAGNOSIS, DIFFERENTIATION, AND THERAPY

Context	Study/ Year	Participants*	ERP
Differentiation	[30] 2019	See in S-Table VIII	**P300**
	[93] 2022	See in S-Table VIII	**P300**
**Total different ERP explored in differentiation**			**1**
Psychotherapy	[94] 2018	61<br>57(4)<br>33.5 (7.9)	**FRN**
	[95] 2018	49 PTSD<br>Not mentioned<br>Range of 18 to 55	**LPP**
	[76] 2019	1 PTSD/23 HC<br>1 Female<br>34 years old	**P3**
	[96] 2021	1 PTSD/100 HC<br>Not mentioned	**P3**
**Total different ERP explored in psychotherapy**			**3**
Pharmacotherapy	[31] 2019	See in S-Table V	**ASS, MMN, P50**
**Total different ERP explored in pharmacotherapy**			**3**

ERP: Event-Related Potentials; LPP: Late Positive Potential; VPP: Vertex Positive Potential; ONE: Old/New Effect ERP ; CNV: Contingent Negative Variation; ENR: Error-Related Negativity ; REWP: Reward Positivity; N100: Negative peak around 100 ms; N170: Negative peak around 170 ms; N200: Negative peak around 200 ms; P100: Positive peak around 100 ms; P300: Positive peak around 300 ms; P3: Another term for P300; NSW: Negative Slow Wave; N1: Negative peak around 100 ms; P2: Positive peak around 200 ms; EPN: Early Posterior Negativity; FRN: Feedback-Related Negativity; ASS: Auditory Stady State; MMN: Auditory Mismatch Negativity.
*M: male(s); F: female(s)

analysis was inconclusive for PTSD identification, this study was classified solely as ERP-based.

According to S-Table VI, there is a noticeable trend towards the use of LPP in PTSD diagnosis, appearing in 7 of 18 studies. Other commonly used potentials are N170, P3 and VPP, each utilized in 3 studies. In addition, there are 17 different ERPs explored and 58.82% of these have been explored once by study. In bold we have highlighted ERPs with the best results among those studied, although studies involving only one ERP have also been highlighted. 63.2% of the studies have only studied one ERP and do not compare it with others. LPP is the ERP most studied. LPP and VPP show the better results in diagnosis, both are positive potentials characterized by positive deflections.

The use of ERPs in differentiation is limited, with only P300 being used in the two studies that addressed this context.

In psychotherapy, P3 (n = 2), FRN (n = 1) and LPP (n = 1) are considered in the assessment of the response to treatments. However, no studies have compared these ERPs against each other or with other ERPs used in the different contexts mentioned. In pharmacotherapy one study uses three ERP and obtains useful results with all of them.

2) *Study Population and Comparison Group*

In this section, we present a detailed analysis of the participants involved in the studies, categorizing them into three distinct groups: PTSD-only participants, healthy control groups, and differentiation groups.

The first group encompasses studies that focus exclusively on PTSD participants, without considering healthy controls or patients with other disorders. These studies primarily address diagnosis, psychotherapy, and pharmacotherapy. Some of these studies further stratify PTSD participants into subgroups based on symptom severity, such as high versus low symptomatology, or by treatment modality. For instance, subgroups may include PTSD patients who received a specific therapy (e.g., EMDR, TDC, TMS) versus those who underwent traditional exposure-based therapy (TEA). The distribution of participants across these contexts is detailed in S-Table VII.

S-TABLE VII
DISTRIBUTION OF PARTICIPANTS ACROSS STUDIES CONSIDERING ONLY PTSD PARTICIPANTS

Studies	Only PTSD	HS PTSD	MS PTSD	LS PTSD
**Diagnosis**				
[81], [85], [88]	✓	-	-	-
[56], [60], [78], [82],	-	✓	-	✓
[59], [61]	-	✓	✓	✓
**Psychotherapy**				
[48], [69], [70], [72], [73], [74], [94], [95], [96], [97], [98]	✓	-	-	-
**Pharmacotherapy**				
[31]	✓	-	-	-

HS: High symptoms; MS: Middle symptoms; LS: Low symptoms.

The second group includes studies that involve healthy control (HC) groups. The remaining diagnosis studies from the 8 studies presented in S-Table VII consider a comparison group with healthy patients. There is only one study in psychotherapy presented by [89] that considers a comparison group with healthy patients. Comprehensive participant details could be seen in the tables of the main manuscript and other tables in supplementary material, in the columns labeled participants.

Finally, we present in the third group studies that include patients with other disorders as a control group. These studies can include a group of healthy patients, but there must necessarily be a group of participants with other disorders. Further details regarding these studies are presented in S-Table VIII.

S-TABLE VIII
DISORDERS COMPARED BY THE STUDIES WHOSE PTSD IDENTIFICATION FOCUSES ON DIFFERENTIATION FROM OTHER DISORDERS

	Study	[30]	[63]	[64]	[65]	[66]	[67]	[93]
Number of Participants by Control Group	**PTSD**	28<br>14 M, 14 F<br>43.50±8.88	12<br>Females<br>No mentioned	40<br>No mentioned<br>30.1±7.9	47<br>See in Table IV	52<br>14 M, 48 F<br>42.74±13	146<br>Not mentioned	30<br>15 M, 15 F<br>42.77±11.16
	**TBI**	-	-	107<br>No mentioned<br>26.7±7.3	-	-	-	-
	**MDD**	67<br>24 M, 43 F<br>42.09±9.83	25<br>Females<br>No mentioned	-	100<br>See in Table IV	-	67<br>Not mentioned	30<br>9 M, 21 F<br>44.60±10.08
	**MOD**	-	-	-	-	226<br>151 M, 115 F<br>30.87±12.70	-	-
	**ANX**	-	-	-	4<br>See in Table IV	107<br>78 M, 28 F<br>29.01±10.56	-	-
	**ADHD**	-	-	-	-	-	95<br>Not mentioned	-
	**BIP**	-	-	-	-	46<br>38 M, 8 F<br>28.48±9.83	81<br>Not mentioned	-
	**OCD**	-	-	-	-		34<br>Not mentioned	-
	**Schizophrenia**	-	-	-	-	117<br>65 M, 62 F<br>31.73±12.10	52<br>Not mentioned	-
	**ADD***	-	-	-	-	186<br>164 M, 22 F<br>29.63±10.89	75<br>Not mentioned	-
	**PNC**	-	-	-	53<br>See in Table IV	59<br>38 M, 21 F<br>31.05±11.30	-	-
	**HC**	39<br>18 M, 21 F<br>38.74±9.05	37<br>Females<br>No mentioned	-	220<br>See in Table IV	95<br>60 M, 35 F<br>25.72 ±4.55	84<br>Not mentioned	-

PTSD: Post-traumatic stress disorder; TBI: Traumatic brain injury; MDD: Major depression disorder; MOD: mood disorder; ANX: anxiety disorders; ADHD: Attention deficit hyperactivity disorder; BIP: Bipolar disorder; OCD: obsessive-compulsive disorder; ADD: addictive disorders; PNC: Panic disorder; HC: healthy controls.

M: Males, F: Females

*Addictive disorders include opioids, general addiction disorders, alcohol use, behavioral addiction.
